# Extended Maxwell equations derived from spacetime geometry

**DOI:** 10.1038/s41598-026-57062-8

**Published:** 2026-06-13

**Authors:** Baoxia Su

**Affiliations:** Department of Applied Physics, Tianjin University, Tianjin, China

**Keywords:** Spacetime product, Extended Maxwell equation, Spacetime curl, Axiomatic derivation, Extended Lorentz vector, Quasi-charge, Astronomy and planetary science, Physics

## Abstract

This paper derives a unified description of electromagnetic and gravitational interactions in the classical regime, addressing the fundamental question of why like charges repel while like masses attract. We formulate a flat spacetime geometry tailored to special relativity and introduce intrinsic operations of spacetime product and spacetime curl, naturally yielding a multi-branched formalism. Based on three axiomatic postulates—relativistic properties of spacetime, energy conservation, and standard charge condition—we rigorously derive four extended Maxwell equations and their corresponding force formulas by employing our formalism, without *ad hoc* parameters or phenomenological fitting. These simultaneously recover electromagnetic and gravitational interactions, along with their mirror-symmetric counterpart, thereby naturally resolving the aforementioned fundamental question. A thought experiment demonstrates consistency with the principle of relativity. We propose a unified charge system to facilitate the unified description. The framework remains strictly classical, does not incorporate spin and radiative-reaction effects, and applies to gravity in quasi-charge conditions where the motion of matter can be treated as a conserved Lorentz four-vector current. This axiomatic derivation provides a geometrically grounded unified description of long-range interactions and a transparent foundation for theoretical extensions.

## Introduction

It is well known that, by analogy^1–5^ to electromagnetism, the theory of gravitoelectromagnetic(GEM)^6–9^ fields and Maxwell-like equations have been formulated for the weak-field limit of gravity in general relativity^1,5,6^. Subsequently some relevant effects^10–16^ were investigated and partially verified experimentally^17,18^. Recently even the strong gravity was described using nonlinear Maxwell equations^19,20^. Apparently, Maxwell equations play a crucial role in both electromagnetism and gravitation, particularly for weak fields in flat spacetime. Maxwell equations can be derived theoretically in two main ways: via Lagrangian mechanics and differential geometry, alongside other approaches^21,22^. Traditionally, Maxwell equation for electromagnetism^23,24^ and Maxwell-like equation for weak gravity^1,2^ can be theoretically obtained in different approaches.

Is it possible to derive Maxwell-like equations and the corresponding interaction forces for both interactions within a single framework? Why are there two distinct interaction systems where like charges repel and like masses attract? These fundamental questions have long attracted wide interest among physics researchers, yet a simple, intuitive, and mathematically rigorous derivation remains lacking.

In this work, we develop a spacetime geometry framework based on foundational mathematical tools—specifically linear algebra and calculus—by introducing spacetime products and Lorentz-covariant spacetime curls. In contrast to GEM theories—which rely on the weak-field linearization of general relativity and formal analogies with electromagnetism—our work adopts a fundamentally distinct axiomatic approach. Grounded in three axiomatic postulates—the relativistic properties of spacetime, standard charge condition, and conservation of energy—our formalism rigorously derives the extended Maxwell equations and the corresponding interaction force equations. We demonstrate that this formulation naturally decomposes into two sectors, corresponding to electromagnetism and gravity respectively. Subsequently, the dynamical features of these two interactions—including their interaction modes (i.e., repulsion between like charges and attraction between like masses), interaction forces, and energy flux density—are derived in a unified manner.

In summary, by employing our spacetime geometry formalism, we have derived the extended Maxwell equations and interaction force formulas from three axiomatic postulates. This formulation characterizes the dynamical features of electromagnetism and gravity, providing definitive answers to the preceding questions. We emphasize that this work is restricted to the classical regime and does not incorporate spin and radiative effects; these limitations are addressed in the Discussion section. Below are some key notations and related backgrounds.

### General notations

Any vector $a\in \mathbb {C}^{3+1}$ will be denoted as $(\mathbf{a}, a_4)$. In complex Minkowski spacetime, we adopt the metric $g_{\mu \nu }=\delta _{\mu \nu } \;(\mu ,\nu =1,2,3,4)$. The square of a vector a is defined as $a^2:= a_{\mu }a_{\mu }=\mathbf{a}\cdot \mathbf{a}+a_4^2$.

### Mathematical framework of spacetime geometry

The curl operator in three-dimensional space plays a pivotal role in Maxwell’s equations, as well as in other fields. Nevertheless, extending this concept to four-dimensional spacetime presents certain challenges. While sophisticated mathematical frameworks such as differential geometry^1^ and Clifford algebra^25–27^ provide effective descriptions of modern physics, they are general-purpose tools not specifically tailored to the structure of 3+1 physical spacetime, and thus do not fully exploit its intrinsic properties to achieve concise formulations of physical laws.

In this work, we develop a specialized spacetime geometry notation designed explicitly for flat 3+1-dimensional relativity,  introducing the intrinsic spacetime product and spacetime curl operations. A notable feature of these constructions is that they admit multiple branches, corresponding to branch properties that arise naturally from the mathematical structure of relativistic spacetime, rather than being imposed by hand.

Owing to its close connection to relativistic spacetime, this formalism offers certain notational and conceptual advantages over standard approaches. In contrast to conventional differential geometry^1^, which requires two independent equations *dF=0* and $d \star F=4\pi \star J$ to express Maxwell’s theory, the spacetime curl operator alone $-i \partial \, \mathbb {R}_{11}\Psi =\textrm{J}/c$ encodes the full set of Maxwell equations. Remarkably, this construction simultaneously yields four distinct versions of Maxwell-type equations $-i \partial \, \mathbb {R}_{\eta \sigma }\Psi =\textrm{J}/c \;(\eta =\pm 1,\sigma =\pm 1$), three of which naturally describe gravitational and mirror-like sectors, allowing us to address fundamental open questions in physics. The example above indicates that the multiple braches of spacetime curls—where each branch possesses a distinct physical significance—highlight the distinct advantage of this notation.

The formalism also extends to other areas of physics and mathematics. In QED, for a spinor *φ*, the equation $\partial _{\mu } \,\textrm{X}_{111,\mu }\,\varphi =0$ represents the massless Dirac equation, consistent with the two-component theory of neutrinos. Within linear algebra, It permits the general eigenvector solution of arbitrary antisymmetric matrices to be expressed in the compact form $x\,\mathbb {X}_{1-11}y$.

In summary, the broad applicability of this spacetime notation across physics and mathematics highlights its deep compatibility with relativistic spacetime and demonstrates clear advantages over conventional mathematical tools within flat relativistic spacetime geometry.

Below is a detailed introduction to “Mathematical framework of spacetime geometry” section. Section Definition of spacetime vector space presents the definition of the spacetime vector space. Section Spacetime products gives the definition of spacetime products, investigates their fundamental properties, and presents their matrix representations. Section Spacetime curls defines spacetime curls and derives several important related identities. In “Incorporation of the mathematical tool into special relativity” section, the above notations are applied to special relativity, and the covariance of spacetime curls is investigated. On this basis, we introduce the definitions of the extended Lorentz vector, special extended Lorentz vector, and relativistic vector, in order to achieve a complete description of all observable physical quantities within the spacetime geometry of relativity. Several corollaries are given, which provide the necessary preparation for the subsequent derivation of the extended Maxwell equations.

### Extended Maxwell equations and unified description

In “Extended Maxwell equations and the unified description” section, First, we introduce several definitions of charge, field, unified charge system and unified description in “Definitions of the unified description” section. Then using the mathematical framework we developed as stated above, we derive the extended Maxwell equations in “Derivation of the extended Maxwell equations” section. Subsequently, formulas of interaction force in “Interaction force and the unified charge unit” section and energy flux in “Energy flux density” section are derived.

### Discussion

In “Discussion” section, we interpret the results presented in “Extended Maxwell equations and the unified description” section, elucidate the physical significance of the chirality and mode properties in the extended Maxwell equations, and discuss the related topics. Section “System with chirality=1 and mode=1 corresponds to electromagnetism” identifies electromagnetism. Section “Chirality property and handedness of the coordinate system” elaborates the relation that chirality property have to the handedness and mirror properties of the system. In “The mode property corresponds to the interaction type of charges” section the mode property *σ* distinguishes between electromagnetism and gravity. Section “Transformation from conventional system to the unified charge system” presents the transformation formulas of the unified charge system. Section “Consistency check via thought experiment” section proposes a thought experiment to verify the internal consistence of unified description. Section “Limitations” presents the limitations of gravity for this formulation. Section “Axiomatic description of electromagnetism and gravitation” presents an axiomatic description of the framework.

## Mathematical framework of spacetime geometry

In this section, we first introduce the definition of a spacetime vector space, then derive its mathematical operations, spacetime product, and spacetime curl, and finally investigate their properties.

### Definition of spacetime vector space

A vector $a:=(\mathbf{a}, a_4) \in \mathbb {C}^{3+1}$ is referred to as a spacetime vector, where $\mathbf{a}\; and\; a_4$ represent its spatial and temporal components, respectively.

#### Definition 1

A spacetime vector space $\textit{V}^{3+1}$ is defined as the set of all spacetime vectors equipped with the usual scalar multiplication and vector addition over complex field.

The space $\textit{V}^{3+1}$ is a complex vector space, and any of the four components can be complex.

### Spacetime products

#### Definition of spacetime products

##### Definition 2

The spacetime product $\mathbb {X}$ is defined as an operation mapping any two spacetime vectors a and b to a spacetime vector c, denoted by $c:=a \,\mathbb {X}\, b$, where $a,b,c \in \textit{V}^{3+1}$ and $c^2=a^2 b^2$.

#### Solutions of spacetime product

We derive the expression for the spacetime product as

1 $$\begin{aligned} a \,\mathbb {X}_{\eta \xi \sigma } \, b = \left( \begin{aligned}&\eta \,\mathbf{a} \times \mathbf{b} + \xi \,\mathbf{a}\,b_4 +\sigma a_4\,\mathbf{b} \\&a_4 b_4 -\xi \sigma \,\mathbf{a} \cdot \mathbf{b} \\ \end{aligned} \right) , \end{aligned}$$

where $\eta =\pm 1,\xi =\pm 1,\sigma =\pm 1$ are referred to as *branch properties* of the spacetime product. For mnemonic purposes, these properties are termed *chirality*, *direction*, and *mode*, respectively. These properties arise naturally during the derivation of the mapping, analogous to the ± sign appearing in front of a radical.

#### Some basic properties of spacetime products.

More properties of the spacetime products are obtained as follows, for $a,b,c \in \textit{V}^{3+1}$:

- The fundamental property by definition : $a \,\mathbb {X}_{\eta \xi \sigma }\, b \, \in \textit{V}^{3+1}\;$, $(a \,\mathbb {X}_{\eta \xi \sigma }\, b)^2 =a^2 \, b^2$.
- The pseudo-commutativity : $a \,\mathbb {X}_{\eta \xi \sigma }\, b = b \,\mathbb {X}_{-\eta \sigma \xi }\, a \,$.
- The bilinearity: $$a \,\mathbb {X}_{\eta \xi \sigma } \,( C_1 \, b + C_2 \,c) =C_1 \,a\,\mathbb {X}_{\eta \xi \sigma }\, b + C_2 \, a\, \mathbb {X}_{\eta \xi \sigma }\, c$$, $$\quad (C_1\,a+C_2\,b) \,\mathbb {X}_{\eta \xi \sigma } \,c =C_1\, a\,\mathbb {X}_{\eta \xi \sigma }\, c + C_2 \,b\, \mathbb {X}_{\eta \xi \sigma }\, c \quad \quad for \;\forall C_1,C_2 \in \mathbb {C}$$.
- For $\xi =\sigma =1$, the algebraic structure $(\textit{V}^{3+1},\mathbb {X}_{\eta 11})$, with the unit vector defined as (0, 0, 0, 1), forms a group.

#### Inverse operation of spacetime products

If there exists a spacetime product $\mathbb {X}_{\eta \xi \sigma }^r$ corresponding to $\mathbb {X}_{\eta \xi \sigma }$ for any two spacetime vectors a and b, satisfying: $a \,\mathbb {X}_{\eta \xi \sigma }^r (a \,\mathbb {X}_{\eta \xi \sigma } \,b) =a^2 \, b$, then $\mathbb {X}_{\eta \xi \sigma }^r$ is referred to as the inverse operation of $\mathbb {X}_{\eta \xi \sigma }$. One can prove that the inverse operation has the properties below:

- The basic property of the inverse operation: $\mathbb {X}_{\eta \xi \sigma }^r = \mathbb {X}_{-\eta (-\xi \sigma )\sigma }$.
- The reciprocity of the inverse operation: $\mathbb {X}_{\eta \xi \sigma }^{rr}=\mathbb {X}_{\eta \xi \sigma }$.

#### Representation matrices of spacetime products

There exists a set of four matrices $\textrm{X}_{\eta \xi \sigma ,\mu } ({\mu }=1,2,3,4)$ corresponding to each $\mathbb {X}_{\eta \xi \sigma }$, satisfying(dropping the subscripts $\eta \xi \sigma$ for simplicity): $a_{\mu } \,\textrm{X}_{\mu }\,b = a \, \mathbb {X} \, b$, for any two spacetime vectors a and b, where $\textrm{X}_{\mu }$ are referred to as the representation matrices of $\mathbb {X}$. One can derive the properties of representation matrices as follows:

- Every matrix of $\textrm{X}_{\mu }({\mu }=1,2,3,4)$ is real orthogonal.
- $\textrm{X}_{\mu }^{-1}$ are representation matrices of $\mathbb {X}^r$.
- Representation matrices satisfy: $\textrm{X}_{\mu }^{-1} \textrm{X}_{\nu }=-\textrm{X}_{\nu }^{-1}\textrm{X}_{\mu } \;\;$ for $\mu \ne \nu$.
- If $a_{\mu }a_{\mu }=1$ then $a_{\mu } \,\textrm{X}_{\mu }$ is orthogonal, and $(a_{\mu } \,\textrm{X}_{\mu })^{-1} = a_{\mu } \,\textrm{X}_{\mu }^{-1}$.

To derive the representation matrices, we employ the two sets of matrices as given below:

$$\begin{aligned} \begin{matrix} \Omega _1=\begin{pmatrix}0& 0& 0& 1\\ 0& 0& -1& 0\\ 0& 1& 0& 0\\ -1& 0& 0& 0\end{pmatrix} & \Omega _2=\begin{pmatrix}0& 0& 1& 0\\ 0& 0& 0& 1\\ -1& 0& 0& 0\\ 0& -1& 0& 0 \end{pmatrix} & \Omega _3=\begin{pmatrix}0& -1& 0& 0\\ 1& 0& 0& 0\\ 0& 0& 0& 1\\ 0& 0& -1& 0 \end{pmatrix}&\Omega _4=I \,, \end{matrix} \end{aligned}$$

$$\begin{matrix} \Theta _1=\begin{pmatrix}0& 0& 0& 1\\ 0& 0& 1& 0\\ 0& -1& 0& 0\\ -1& 0& 0& 0 \end{pmatrix} & \Theta _2=\begin{pmatrix}0& 0& -1& 0\\ 0& 0& 0& 1\\ 1& 0& 0& 0\\ 0& -1& 0& 0 \end{pmatrix} & \Theta _3=\begin{pmatrix}0& 1& 0& 0\\ -1& 0& 0& 0\\ 0& 0& 0& 1\\ 0& 0& -1& 0 \end{pmatrix}&\Theta _4=I \,. \end{matrix}$$

For ${\mu }=1,2,3,4$, we can prove:

2 $$\begin{aligned} \textrm{X}_{111,\mu } = \Omega _{\mu } \,, \quad \textrm{X}_{-1-11,\mu } = \Omega _{\mu }^{-1} \,, \quad \textrm{X}_{-111,\mu } = \Theta _{\mu } \,, \quad \textrm{X}_{1-11,\mu } =\Theta _{\mu }^{-1} \,, \quad \textrm{X}_{\eta \xi -1,\mu } = \textrm{I}'\,\textrm{X}_{-\eta -\xi 1,\mu } \,, \end{aligned}$$

where $$\textrm{I}' = diag(-1,-1,-1,1)$$.

### Spacetime curls

With the vector operator $\partial :=(\nabla ,\partial _4)$, the operation $\partial \,\mathbb {X}_{\eta \xi \sigma }$ acting on the spacetime vector $\phi =(\boldsymbol{\phi },\phi _4)$ as in Eq. (1) is referred to as *the spacetime derivative*. $\partial \,\mathbb {X}_{\eta \xi \sigma }$ would be a mathematically 4-dimensional curl, if for any *ϕ* it satisfies

3 $$\begin{aligned} \partial \cdot \partial \,\mathbb {X}_{\eta \xi \sigma } \,\phi =0\,. \end{aligned}$$

However, No $\partial \,\mathbb {X}_{\eta \,\xi \,\sigma }$ satisfies the above equation strictly, except that $\partial \,\mathbb {X}_{\eta \,1\,\sigma }$ satisfies Eq. (3) conditionally. We denote the four of the eight $\mathbb {X}_{\eta \xi \sigma }$ by $\mathbb {R}_{\eta \sigma }:= \mathbb {X}_{\eta 1\sigma }$, which can be extended to be curls in the following subsection.

#### Definition of spacetime curls

##### Definition 3

The operation $\partial \,\mathbb {R}_{\eta \sigma }$ is referred to as the spacetime curl if it acts on the spacetime vector $\phi =(\boldsymbol{\phi },\phi _4)$ as follows:

4 $$\begin{aligned} \partial \,\mathbb {R}_{\eta \sigma } \,\phi := \left( \begin{aligned}&\eta \,\nabla \times \boldsymbol{\phi } + \nabla \, \phi _4 +\sigma \partial _4\,\boldsymbol{\phi } \\&\partial _4 \phi _4 -\sigma \, \nabla \cdot \boldsymbol{\phi } \end{aligned} \right) , \end{aligned}$$

where $\mathbb {R}_{\eta \sigma }:= \mathbb {X}_{\eta 1\sigma }$. Evidently, there esist four spacetime curls. The equation $\partial \,\mathbb {R}_{\eta \sigma } \,\phi =\psi$ is referred to as the spacetime curl equation, which can be expressed in matrix form as $\partial _{\mu }\textrm{R}_{\eta \sigma ,\mu } \phi =\psi$ where

5 $$\begin{aligned} \textrm{R}_{\eta \sigma ,\mu } := \textrm{X}_{\eta 1\sigma ,\mu } \end{aligned}$$

is the representation matrix of $\mathbb {R}_{\eta \sigma }$, and $\textrm{X}_{\eta 1\sigma ,\mu }$ can be obtained from Eq. (2). Below are several useful formulas:

6 $$\begin{aligned} \mathbb {R}_{\eta \sigma }^r= \mathbb {X}_{-\eta -\sigma \sigma } \end{aligned}$$

7 $$\begin{aligned} \partial \cdot \partial \,\mathbb {R}_{\eta \sigma } \,\phi = \square \phi _4 \end{aligned}$$

8 $$\begin{aligned} \partial \,\mathbb {R}_{\eta \sigma }(\partial \,\mathbb {R}_{\eta \sigma }^r \phi ) = \partial \,\mathbb {R}^r_{\eta \sigma }(\partial \,\mathbb {R}_{\eta \sigma } \phi ) =\square \phi \end{aligned}$$

### Incorporation of the mathematical tool into special relativity

With the mathematical tools fully elaborated above, we herein integrate them with special relativity to construct the basic notions for the treatment of classical fields. Note that Lorentz four-vector coordinates read x:= (x_1_, x_2_, x_3_, *ict*), and its square acts as a Lorentz scalar in special relativity.

We adopt the spacetime vector to represent a physical quantity if the quantity meets the following criterion: under SO(3) coordinate rotations, it transforms in the same manner as a Lorentz vector. Since nearly all observable three-dimensional physical quantities (with the fourth component set to zero) and four-dimensional physical quantities fulfill this requirement, identifying them as spacetime vectors is a natural correspondence. Especially, when the transformation of the vector of a observable is unknown, it can be a spacetime vector naturally.

Considering that the frame of reference *x'y'z'* moves with velocity $\mathbf{v}$ relative to that of *xyz*, the Lorentz transformation matrix $\textrm{L}$ is given by^23,24^

9 $$\begin{aligned} \textrm{L}=\begin{pmatrix} 1+\kappa \textrm{u}_1^2 & \kappa \textrm{u}_1\textrm{u}_2 & \kappa \textrm{u}_1 \textrm{u}_3 & i\beta \gamma \textrm{u}_1\\ \kappa \textrm{u}_2\textrm{u}_1 & 1+\kappa \textrm{u}_2^2 & \kappa \textrm{u}_2\textrm{u}_3 & i\beta \gamma \textrm{u}_2\\ \kappa \textrm{u}_3\textrm{u}_1 & \kappa \textrm{u}_3\textrm{u}_2 & 1+\kappa \textrm{u}_3^2 & i\beta \gamma \textrm{u}_3\\ -i\beta \gamma \textrm{u}_1 & -i\beta \gamma \textrm{u}_2 & -i\beta \gamma \textrm{u}_3 & \gamma \\ \end{pmatrix}, \end{aligned}$$

where $$\textrm{u} \equiv \mathbf{v}/ \Vert \mathbf{v}\Vert , \, \beta \equiv \Vert \mathbf{v}\Vert /c, \, \gamma \equiv 1/\sqrt{1-\beta ^2} \; and \; \kappa \equiv \gamma -1$$.

#### Definition 4

A spacetime vector *ϕ* is referred to as an extended Lorentz vector if, under a Lorentz transformation, it transforms as $\phi \mapsto \mathbf{T} \phi$ , where $\mathbf{T}$ is an orthogonal matrix.

#### Theorem 1

*Let* *ϕ* *be an extended Lorentz vector and* *ψ* *a Lorentz vector. A necessary and sufficient condition for the spacetime curl equation*

10 $$\begin{aligned} \partial _{\mu }\textrm{R}_{\eta \sigma ,\mu } \phi =\psi \end{aligned}$$

*to be Lorentz covariant is that the transformation matrix of* *ϕ* *under the Lorentz transformation Eq.* (9) *is given by*

11 $$\begin{aligned} \textrm{T}= \begin{pmatrix} & & & 0 \\ & \mathbf{T} & & 0 \\ & & & 0 \\ 0 & 0 & 0 & 1 \\ \end{pmatrix}, \end{aligned}$$

*where the upper left* *3× 3* *sub-matrix* $\mathbf{T}$ *is given by*

12 $$\begin{aligned} \mathbf{T} = \textrm{U}_0 + i \eta \sigma \textrm{V}_0 , \end{aligned}$$

*where*

13 $$\begin{aligned} \left\{ \begin{aligned}&\textrm{U}_0 = \gamma \textrm{I} - \kappa {{\textbf {u}}} {{\textbf {u}}} \\&\textrm{V}_0 = \beta \gamma \begin{pmatrix} 0 & \textrm{u}_3 & -\textrm{u}_2 \\ -\textrm{u}_3 & 0 & \textrm{u}_1 \\ \textrm{u}_2 & -\textrm{u}_1 & 0 \\ \end{pmatrix} \end{aligned} \right. , \end{aligned}$$

*where* ${{\textbf {u}}} {{\textbf {u}}}$ *in the first line of the preceding equation represents a dyadic matrix.*

#### Definition 5

A special extended Lorentz vector is defined as an extended Lorentz vector whoes transformation matrix is Eq. (11) under the Lorentz transformation Eq. (9).

#### Corollary 1

*For the special extended Lorentz vector* $\phi =(\boldsymbol{\phi },\phi _4),$ *the* $\phi _4$ *and* $\phi \cdot \phi$ *are both Lorentz scalars.*

#### Corollary 2

*For any continuous observable Lorentz-vector quantity* *ψ,* *we can define a differentiable special extended Lorentz vector* *ϕ* *such that Eqs.* (10) *and* (11) *in Theorem* 1*are satisfied.*

#### Corollary 3

*For a special extended Lorentz vector* *ϕ,* *there exists a Lorentz vector* *θ* *such that* $\phi = \partial \, \mathbb {R}_{\eta \sigma }^r \theta$.

#### Definition 6

A spacetime vector is referred to as a relativistic vector if its square is Lorentz scalar.

These definitions are introduced to rigorously characterize relativistic physical quantities within our notational framework, while the accompanying corollaries ensure logical clarity and traceability in physical applications.

Apparently, a scalar s can be extended to be a special extended Lorentz vector (0, 0, 0, *s*), according to Corollary 1. It is evident that both Lorentz vectors and extended Lorentz vectors belong to the class of relativistic vectors, The energy flux density $(\mathbf{E}\times \mathbf{B}, i(\mathbf{E}^2 + \mathbf{B}^2)/2)$ is a relativistic vector, whereas it is not an extended Lorentz vector. The set of relativistic vectors constitutes the largest family of vectors associated with special relativity.

## Extended Maxwell equations and the unified description

We attempt to derive the extended Maxwell equations and explore its physical application within this mathematical framework by employing the four spacetime curl equations.

### Definitions of the unified description

For the sake of theoretical integration and rigor, we present the following general definitions. *Physical quantities that induce interactions between two spatially separated objects are termed* *charges*, *while those that mediate such interactions through space are referred to as* *fields*. Thus the mass associated with gravitation is now referred to as charge—or more specifically, gravitational charge. Since both electric charges and gravitational charges give rise to inverse-square forces, they ought to share the same dimension and unit. Hereafter, physical quantities including charges, currents, fields, and potentials—along with their corresponding formulas—are redefined to unify their dimensions and units for both electromagnetism and gravity. We may temporarily refer to such a system as a unified charge system or unified charge dimension. If not specified these quantities can refer to either electromagnetic or gravitational ones. For example, the current density denoted by Lorentz vectors $(\mathbf{J},i c\rho )$ can be either electric current density or gravitational current density. The specific dimension and unit of some physical quantities will be given later in this section.

We define the **standard** **charge condition** by the following two requirements: (1) the flow of charge is described by a Lorentz four-vector current; and (2) the corresponding current is locally conserved. Any charge that fulfills the above condition is defined as a standard charge. In contrast, charges that do not satisfy the condition strictly but meet it approximately under specific constraints are defined as **quasi-charges**, and the relevant constraints are termed **quasi-charge condition**, which is specifically given in “Limitations” section. The derivations presented below hold exactly for standard charges and approximately for quasi-charges. Gravitational charges fall into the category of quasi-charges, and the gravitational interaction discussed in this work complies with the quasi-charge condition. The general limitations of gravitational effects will be addressed in the next section.

### Derivation of the extended Maxwell equations

Under the standard charge condition (1), for the Lorentz four-vector $\textrm{J} = (\mathbf{J},ic\rho )$, we define, in accordance with Corollary 2, the special extended Lorentz vector $\Psi =(\mathbf {\Psi },\Psi _4)$ that satisfies the following covariant equation:

14 $$\begin{aligned} -i \partial \, \mathbb {R}_{\eta \sigma }\Psi =4{\pi}\textrm{J}/c, \end{aligned}$$

where $\eta =\pm 1,\sigma =\pm 1$, *c* is the speed of light, and *-i* is introduced for consistency with the convention in classical field theory. The *Ψ* can be interpreted as the field induced by the source current density $\textrm{J}$. According to Corollary 1, $\Psi _4$ is Lorentz invariant. Taking the 4-divergence on both sides of Eq. (14),  under the standard charge condition (2),  namely,  current conservation $\partial _{\mu }\textrm{J}_{\mu }=0$, by virtue of Eq. (7) we obtain

15 $$\begin{aligned} \square \Psi _4=0, \end{aligned}$$

which is referred to as the additive condition to Eq. (14). We have now derived, from first principles, the initial differential equations (14) and the additive condition (15), which describe the relationship between the current density and its induced fields. Next we will analyze and exploit these equations in detail.

According to Corollary 3, the field *Ψ*, which is a special extended Lorentz vector, can be expressed in terms of a Lorentz vector $\textrm{A}$ as

16 $$\begin{aligned} \Psi = -i \partial \, \mathbb {R}_{\eta \sigma }^r \textrm{A}. \end{aligned}$$

Substituting *Ψ* in Eq. (16) into (14) and using Eq. (8), we get

17 $$\begin{aligned} \square \textrm{A}=-4{\pi}\textrm{J}/c . \end{aligned}$$

The vector $\textrm{A}$ appearing in Eqs. (16) and (17) is identified as a Lorentz vector potential, since Eq. (17) closely mirrors the well-known equation connecting the Lorentz vector potential and current density in classical field theory^23,24^. Subsequently, applying the Lorentz condition $\partial _{\mu } \textrm{A}_{\mu }=0$ to Eq. (16), we obtain $\Psi _4=0$, which fulfils the additive condition (15). Thus *Ψ* can be rewritten as

18 $$\begin{aligned} \Psi = \begin{pmatrix}\mathbf{E}+i\mathbf{B} \\ 0 \end{pmatrix}, \end{aligned}$$

where $\mathbf{E}$ and $\mathbf{B}$ are the real and imaginary parts of $\mathbf {\Psi }$, respectively. Rewriting Eq. (14) in terms of $\mathbf {\Psi }$ and in the matrix form of *Ψ*, respectively, we obtain the following two types of equations:

19 $$\begin{aligned} \left\{ \begin{aligned}&-i\eta \nabla \times \mathbf {\Psi } - \sigma \dfrac{\partial \mathbf {\Psi }}{c\,\partial t}= 4{\pi}\mathbf{J}/c \\&\sigma \nabla \cdot \mathbf {\Psi } =4{\pi}\rho \end{aligned} \right. \end{aligned}$$

20 $$\begin{aligned} -i \partial _{\mu }\, \textrm{R}_{\eta \sigma ,\mu } \, \Psi =4{\pi}\textrm{J}/c, \end{aligned}$$

where the matrices $\textrm{R}_{\eta \sigma ,\mu }$ can be obtained from Eqs. (5) and (2).

Substituting *Ψ* in Eq. (18) into Eq. (14) and using Eq. (4), we obtain the extended Maxwell equations in real form as

21 $$\begin{aligned} \left\{ \begin{aligned}&\eta \nabla \times \mathbf{E} + \sigma \dfrac{\partial \mathbf{B}}{c\,\partial t}= 0 \\&\eta \nabla \times \mathbf{B} - \sigma \dfrac{\partial \mathbf{E}}{c\,\partial t} = 4{\pi}\mathbf{J}/c \\&\sigma \nabla \cdot \mathbf{B} = 0 \\&\sigma \nabla \cdot \mathbf{E} =4{\pi}\rho , \end{aligned} \right. \end{aligned}$$

where $\eta =\pm 1$ and $\sigma =\pm 1$ denote the chirality and mode of the extended Maxwell equations, respectively, inherited from the spacetime product defined in Eq. (1). Equations (14),(19), and (20) can also be referred to as *the extended Maxwell equations* in different mathematical forms, respectively. The extended Maxwell equations above contain four distinct sets of Maxwell equations for different values of the properties *η* and *σ*. The conventional Maxwell equations are restored when *η = 1* and *σ = 1*. The remaining three sets of Maxwell equations, which are covariant, are referred to as covariant isomers of the conventional Maxwell equations, borrowing the term from chemistry.

According to Corollary 1, we get two frequently used Lorentz scalars in complex form as $\Psi \cdot \Psi =\mathbf{E}^2-\mathbf{B}^2+2i\mathbf{E}\cdot \mathbf{B} \,$. For vacuum state $\textrm{J}=0$, with $\partial \,\mathbb {R}^{r}_{\eta \sigma }$ operating on Eq. (14) and by virtue of Eq. (8) one can readily obtain the Helmholtz equation $\square { \Psi } = 0$, which also demonstrates the simplicity that the spcetime curl brings to us.

### Interaction force and the unified charge unit

In this subsection, based on the principle of energy conservation, we derive the force density f exerted on a current $\textrm{J}$ in the corresponding field within the special relativity framework. From the laws of special relativistic dynamics and field energy conservation, the relation between the two Lorentz four-vectors is readily derived as

22 $$\begin{aligned} \textrm{f} \cdot \textrm{J} =0 \end{aligned}$$

From Eq. (22) and basic linear algebra, we know that there exists an antisymmetric matrix relating the two vectors f and $\textrm{J}$. Since the two vectors are Lorentz vectors, this antisymmetric matrix is identified to be the antisymmetric tensor of the field. In this way, we obtain the expression for f as follows:

23 $$\begin{aligned} \textrm{f} = \zeta \textrm{KJ}/c. \end{aligned}$$

where, from Eq. (21), the antisymmetric tensor $\textrm{K}$ is obtained as follows

24 $$\begin{aligned} \textrm{K}=\begin{pmatrix} 0 & \eta \textrm{B}_3 & -\eta \textrm{B}_2 & -i\sigma \textrm{E}_1\\ -\eta \textrm{B}_3 & 0 & \eta \textrm{B}_1 & -i\sigma \textrm{E}_2\\ \eta \textrm{B}_2 & -\eta \textrm{B}_1 & 0 & -i\sigma \textrm{E}_3\\ i\sigma \textrm{E}_1 & i\sigma \textrm{E}_2 & i\sigma \textrm{E}_3 & 0 \\ \end{pmatrix} \end{aligned}$$

and the Lorentz-invariant coefficient *ζ* is a function of *η* and *σ*, but presently unknown to us. Substituting Eq. (24) and $\textrm{J}=(\mathbf{J},ic\rho )$ into Eq. (23) we get

25 $$\begin{aligned} \textrm{f}=\zeta \begin{pmatrix} \sigma \rho \mathbf{E}+\eta \mathbf{J} \times \mathbf{B}/c \\ i \sigma \mathbf{E} \cdot \mathbf{J}/c \end{pmatrix} \end{aligned}$$

(Conventionally, the static field $\mathbf{E}$ is determined from the measured force $\mathbf{F}$ acting on a test charge *q* placed in the field $\mathbf{E}$, defined as $\mathbf{E}:=\mathbf{F}/q$.) Since the force density $\,\mathbf{f} = \zeta \sigma \rho \mathbf{E}$ when $\mathbf{B}=0$, a comparison with the conventional definition $\mathbf{E}:=\mathbf{f}/\rho$ yields $\zeta =\sigma$. Substitute *ζ* into Eq. (25), we obtain the force density:

26 $$\begin{aligned} \textrm{f}=\begin{pmatrix} \rho \mathbf{E}+ \eta \sigma \mathbf{J} \times \mathbf{B}/c \\ i \mathbf{E} \cdot \mathbf{J}/c \end{pmatrix} \end{aligned}$$

Taking integral of the forth line of Eq. (21) and the $\rho \mathbf{E}$ item of Eq. (26), we get the force that the charge $Q_1$ exerts on $Q_2$ in stationary state as:

27 $$\begin{aligned} \mathbf{F}_{12}=\sigma \frac{\textrm{Q}_1\textrm{Q}_2}{\textrm{r}^2}\mathbf{e}_{12} \end{aligned}$$

Equation (27) is the universal formula for calculating the interaction forces of the four systems in the unified description with mode property $\sigma =\pm 1$. The unit of charge in the unified charge system is $Newton^{1/2}\cdot meter$ , which means that the magnitude of the interaction force between two point charges, separated by one meter and each carrying one unit of electric charge, is one newton. The units of field $\mathbf{E}$ and potential $\mathbf{A}$ are $Newton^{1/2}/meter$ and $Newton^{1/2}$ respectively. By a similar derivation,we obtain the formula for the interaction forces between two current elements^16^ as follows

28 $$\begin{aligned} d\mathbf{F}_{12}=\sigma \frac{\textrm{I}_1\textrm{I}_2 d\mathbf{l}_2\times (d\mathbf{l}_1\times \mathbf{e}_{12}) }{c^2\textrm{r}^2} \end{aligned}$$

### Energy flux density

The energy flux density, in analogy to that in Electrodynamics^23^, is defined as

29 $$\begin{aligned} \textrm{S} := c(\Psi ^*\,\mathbb {R}_{\eta \sigma } \Psi )/(8{\pi}i), \end{aligned}$$

which is the expansion of the Poynting vector. Substituting Eq. (18) into the above equation, one gets

30 $$\begin{aligned} \textrm{S} =c\begin{pmatrix}\eta \mathbf{E}\times \mathbf{B} \\ i\sigma (\mathbf{E}^2 + \mathbf{B}^2)/2 \end{pmatrix}/(4{\pi}) . \end{aligned}$$

By virtue of Eq. (21) we get $\partial _{\mu } \textrm{S}_{\mu } = -\mathbf{E}\cdot \mathbf{J}$. With the help of Eq. (26), one will get $\partial _{\mu } \textrm{S}_{\mu } = -\mathbf{f}\cdot \mathbf{v}$, which complies with the law of energy conservation. One can prove $\textrm{S}$ is a relativistic vector, since from Eq. (29) one can obtain:

31 $$\begin{aligned} \textrm{S}^2 =-c^2 |\Psi ^2 |^2/(64{\pi}^2) \end{aligned}$$

## Discussion

In “Extended Maxwell equations and the unified description” section, we derived the extended Maxwell equations, interaction force formulas, and other relevant expressions, all of which incorporate the branch properties *η* and *σ*. In fact, these parameters are inherited from the spacetime curls, which inherently reflect the fundamental characteristics of spacetime. In this section, we proceed to discuss the physical significance of each branch property and give a validation of the unified description.

### System with chirality=1 and mode=1 corresponds to electromagnetism

Substituting *η =1* (chirality) and *σ =1* (mode) into Eqs. (21) and (20), we get the conventional Maxwell equation. The conventional Maxwell equation in complex form of the system are below

32 $$\begin{aligned} -i \partial _{\mu } \,\Omega _{\mu } \Psi =4{\pi}\textrm{J}/c \end{aligned}$$

All other derived formulas are consistent with the conventional theory of electromagnetism^24^. Note that in the unified charge system, the $\mathbf{E}$ and $\mathbf{B}$ in Eqs. (18) and (21) etc are calculated in the same dimension and unit.

### Chirality property and handedness of the coordinate system

Observing Eqs. (21),(26) and (30), one can find that the chirality property *η* always appears near the cross-product operator,which means that the right-hand system with *η =1* and the left-hand system with *η =-1* are equivalent. Accordingly, the chirality property *η* relates to the handedness of the coordinate system. When describing the electromagnetism and gravity, you should set *η =1* if the system is right-handed, and *η =-1* if left-handed. Otherwise the system represents a mirror one.

### The mode property corresponds to the interaction type of charges

From Eqs. (27) and (28),we show that the mode property *σ =1* corresponds to the repulsion between like charges and attraction between opposite charges, whereas *σ =-1* corresponds to the attraction between like charges and repulsion between opposite charges. Thus the mode property *σ* is also referred to as the mode property of charges and their corresponding interactions, where *σ =1* corresponds to electromagnetism, and *σ =-1* to gravitation. Therefore, we theoretically derive that like charges (σ = 1) repel while like masses (σ = −1) attract.

### Transformation from conventional system to the unified charge system

In “Extended Maxwell equations and the unified description” section, One of the reasons we can naturally describe electromagnetic and gravitational interactions using a concise and unified set of formulas lies in our adoption of the unified charge system. Below, we present how the relevant physical quantities in this system are converted from their counterparts in the conventional SI unit system. Specificly the current density $\textrm{J}= (\mathbf{J},i c\rho )$ in the unified charge system can be transformed from the conventional current density $\textrm{J}_{t}=(\mathbf{J}_{t},i c\rho _{t})$ as follows:

33 $$\begin{aligned} \textrm{J}=\textrm{C}_t \textrm{J}_{t}, \end{aligned}$$

where for electromagnetism $\textrm{C}_t=1/\sqrt{4\pi \epsilon _0}$, $\textrm{J}_{t}$ is conventional electrical current density; for gravity $\textrm{C}_t=\sqrt{G}$, $\textrm{J}_{t}$ is the mass current density, where the mass is rest mass and is Lorentz scalar. The transformation formulas for charges, fields and potentials can be readily derived by analogy with Eq. (33).

### Consistency check via thought experiment

This section presents a thought experiment designed to perform a consistency check of the extended Maxwell equations and force formulas for both electromagnetic and gravitational interactions with the principle of relativity.

We assume that there exist two stationary objects $m_1$ and $m_2$ in outer space, carrying electric charges $q_1$ and $q_2$ of the same sign, which satisfy $q_1\,q_2/(4\pi \epsilon _0)= G m_1\,m_2$. This leads to the cancellation of the two forces in Eq. (27). One will observe that their interaction vanishes in the rest frame of the objects. By the principle of relativity, one should also observe the vanishing interaction—including the magnetic-like force—in the moving frame. We can readily demonstrate that the unified description satisfies the above description, as derived from Eqs. (21) (27) and (28). Conversely, if gravity does not conform to the unified description, the principle of relativity cannot be satisfied.

For example, assuming that the magnetic-like force in gravity does not exist or is weaker than predicted by the unified description, the cancellation of forces in the moving frame would no longer hold. In that case, observers in the moving frame would detect relative motion between the two objects, whereas those in the rest frame would still observe the objects to remain stationary. The two frames would thus observe distinct physical phenomena, which would violate the principle of relativity.

In summary, this thought experiment shows that the extended Maxwell equations and interaction force formulas for the two interactions yield consistent and well‑defined predictions. Importantly, this thought experiment does not serve as experimental validation distinguishing the present framework from standard GEM theories, but instead verifies that the formulation is consistent with the principle of relativity. Essentially, the validity of this thought experiment implies an internal coherence between the two interactions, dictated by the principle of relativity. The consistency check indicates that our formulation for the two interactions satisfies the requirement of internal coherence. Specifically, this is manifested in the symmetry of the force expressions and the covariance of the forces.

### Limitations

A key limitation of this framework is that it is confined to the classical regime and does not incorporate radiation or spin effects. For gravitational applications, additional conditions must be satisfied for the formulation to hold—these are referred to as the quasi-charge condition as defined in “Definitions of the unified description” section. Specifically, the condition is fulfilled when the motion of the source matter can be characterized by a conserved Lorentz vector current, which requires the sources to be slowly moving, free of internal interactions, non-relativistic (no relative motion), and distant (i.e., in the weak-field regime). While these constraints may appear stringent, representative examples that roughly satisfy them include non-rotating rigid bodies and uniformly moving dilute media. By implementing appropriate approximations based on these conditions, selecting a suitable gauge, and performing linearization, Einstein’s field equations can be reduced to one of the extended Maxwell equations ($\textrm{mode}=-1$); this set of operations is termed the quasi-charge approximation.

Notably, the gravitomagnetic force between current elements Eq. (28) takes a form analogous to its electromagnetic counterpart, and represents one level of approximation within the quasi-charge approximation. However, its numerical coefficients may differ from those in full linearized general relativity (GR), arising from discrepancies in gauge choices and approximation schemes. For precise coefficient values, we refer the reader to established standard literature on gravitational electromagnetism (GEM)^6,10^.

### Axiomatic description of electromagnetism and gravitation

To recap the preceding derivations and discussions within an axiomatic framework, we formulate four postulates and carry out an axiomatic derivation of the corresponding results as follows.

#### Postulate 1

All physical observables can be represented as Lorentz vectors, extended Lorentz vectors, relativistic vectors or spacetime vectors, and the formulations of all physical laws are form-invariant under the Lorentz group.

#### Postulate 2

The dynamics of matter in spacetime are governed by the principles of special relativity.

#### Postulate 3

The principle of energy conservation holds.

#### Postulate 4

The charges under consideration satisfy *the standard charge condition*, where the charge flow forms a locally conserved Lorentz four-vector current.

The existence and fundamental properties of electromagnetism and gravity can be derived from the four preceding postulates. As an illustration, consider two charged objects separated in space. The definition of charge entails the existence of an interaction, whereas Postulate 2 precludes action at a distance. Consequently, we infer that fields must exist to mediate such interactions, which are sourced by the current described in Postulate 4. Since these results, together with Postulate 3 and 1, fulfill all the conditions required in “Extended Maxwell equations and the unified description” section, both the extended Maxwell equations (21) and the interaction force formula (26) are thus derived simultaneously. The results indicate that there exist two distinct types of interactions, which recover electromagnetism and gravitation in both the (Maxwell) field equations and the corresponding interaction forces.

Notably, sometimes for simplicity, Postulate 1 and 2 are collectively referred to as the relativistic properties of spacetime. As stated in “Incorporation of the mathematical tool into special relativity” section, scalars can be expressed as special extended Lorentz vectors for Postulate 1 .

## Conclusions

In “Mathematical framework of spacetime geometry” section, we establish a flat spacetime geometry for special relativity, introducing the operations of spacetime product and spacetime curl. This formalism is characterized by a concise mathematical form and the natural emergence of multiple branches, which arise from rigorously derivations rather than artificial imposition. To describe physical quantities within this framework, we define extended Lorentz and relativistic vectors, and derive a theorem and several corollaries regarding the spacetime curl.

In “Extended Maxwell equations and the unified description” section, based on the axiomatic postulates of relativistic spacetime properties, standard charge condition, and energy conservation, we rigorously derived four sets of extended Maxwell’s equations and their corresponding force expressions. These describe electromagnetism, gravity, and their respective mirror images.

Notably, (1) The consistency of this theory with the principle of relativity has been verified via a thought experiment. (2) To achieve conciseness and symmetry in the formulations of the two interactions, we have introduced a unified charge system. (3) The theory is applicable to the classical regime and does not incorporate spin or radiation. (4) For the case of gravity, the theory requires the quasi-charge condition to be satisfied.

In conclusion, our formalism yields the results based on three axiomatic postulates, without relying on subjective assumptions or parameter tuning. We show that the existence of two distinct interaction modes (repulsion of like charges vs. attraction of like masses) is dictated by the relativistic properties of spacetime and energy conservation. These findings imply that the dynamical features of long-range interactions are governed by spacetime structure and conservation laws.

By integrating our framework with existing theories, we can categorize the study of the interactions into two distinct approaches: (1) In the geometric-dynamical and spacetime-based approach, our theory is formulated via extended Maxwell equations and a unified interaction force law, distinguished by the parameter mode ($\textrm{mode}=1$ for electromagnetism, $\textrm{mode}=-1$ for gravity), as detailed in Eq. (21) and “The mode property corresponds to the interaction type of charges” section. The similarities and differences between the two interactions are classified and governed by relativistic spacetime properties. (2) In the field-theoretic and symmetry-based approach, standard modern physics employs distinct Lagrangians, distinguishing the interactions by spin (spin-1 and spin-2), with their physical properties dictated by internal symmetries such as gauge invariance and diffeomorphism invariance.

By recasting the fundamental questions within this mathematical framework, our approach offers a novel foundational perspective on the two interactions. Several open questions remain for its further development, including establishing a rigorous correspondence with standard spin- and symmetry-driven formulations, and investigating the physical limits under which the two descriptions converge or map onto each other. Future work along these lines may deepen the unified understanding of electromagnetic and gravitational interactions across geometric and quantum frameworks.

## Data Availability

All data generated or analysed during this study are included in this published article.
